# Detection of hepatitis C virus (HCV) negative strand RNA and NS3 protein in peripheral blood mononuclear cells (PBMC): CD3^+^, CD14^+^ and CD19^+^

**DOI:** 10.1186/1743-422X-10-346

**Published:** 2013-11-26

**Authors:** Agnieszka Pawełczyk, Natalia Kubisa, Joanna Jabłońska, Iwona Bukowska-Ośko, Kamile Caraballo Cortes, Maria Fic, Tomasz Laskus, Marek Radkowski

**Affiliations:** 1 Department of Immunopathology of Infectious and Parasitic Diseases, Warsaw Medical University, Warsaw, Poland; 2 Department of Hepatology and Acquired Immune Deficiencies, Warsaw Medical University, Warsaw, Poland

**Keywords:** HCV, PBMC, Extrahepatic replication, NS3 protein, Negative strand HCV RNA

## Abstract

**Background:**

Although hepatitis C virus (HCV) is primarily hepatotropic, markers of HCV replication were detected in peripheral blood mononuclear cells (PBMC) as well as in *ex vivo* collected tissues and organs. Specific strains of HCV were found to be capable to infect cells of the immune system: T and B cells and monocytes/macrophages as well as cell lines *in vitro*. The direct invasion of cells of the immune system by the virus may be responsible for extrahepatic consequences of HCV infection: cryoglobulinemia and non-Hodgkin’s lymphoma.

The aim of the present study was to determine the prevalence of markers of HCV infection: negative strand HCV RNA and non-structural NS3 protein in PBMC subpopulations: CD3^+^, CD14^+^ and CD19^+^. The presence of virus and the proportion of affected cells within a particular PBMC fraction could indicate a principal target cell susceptible for HCV.

**Methods:**

PBMC samples were collected from 26 treatment-free patients chronically infected with HCV. PBMC subpopulations: CD3^+^, CD14^+^, CD19^+^ were obtained using positive magnetic separation. The presence of negative strand RNA HCV and viral NS3 protein were analyzed by strand-specific RT-PCR and NS3 immunocytochemistry staining.

**Results:**

Negative strand HCV RNA was detectable in 7/26 (27%), whereas NS3 protein in 15/26 (57.6%) of PBMC samples. At least one replication marker was found in 13/26 (50%) of CD3^+^ cells then in 8/26 (30.8%) of CD14^+^ and CD19^+^ cells. The highest percentage of cells harboring viral markers in single specimen was also observed in CD3^+^ (2.4%), then in CD19^+^ (1.2%), and much lower in CD14^+^ (0.4%) cells.

**Conclusions:**

Our results indicate that CD3^+^ cells are a dominant site for extrahepatic HCV replication, although other PBMC subpopulations may also support virus replication.

## Background

Multiple studies showed that hepatitis C virus (HCV) is not a strictly hepatotropic pathogen [1-4]. In recent years, the evidence of the extrahepatic HCV replication by detection of positive and negative strand HCV RNA and viral proteins were shown in peripheral blood mononuclear cells (PBMC); [5-8], bone marrow [9] and in the central nervous system [10,11] as well as *in vitro* in cell culture models: macrophages, cell lines MT2, Huh7, Jukat, Molt-4 [12].

Molecular markers of extrahepatic HCV replication were detected frequently in subjects with severely impaired immunity, in particular due to advanced HIV infection [2,3,5,13]. In these patients cells supporting HCV replication were T and B lymphocytes and monocytes/macrophages [6,14].

The clinical consequences of HCV extrahepatic replication are known, as the role of viral infection in the induction of type II mixed cryoglobulinemia and B-cell non-Hodgkin lymphoma (B-NHL) is well ascertained [15-18]. However, the pathogenetic mechanisms underlying these phenomena are poorly understood. They may include functional defects, abnormal proliferation and differentiation of lymphocytes due to chronic inflammatory process and/or direct cell infection by the virus [19-22].

To characterize the extrahepatic sites of HCV replication and virus reservoirs in the organism the studies of separate markers of HCV replication in PBMC were performed [2,8,23,24], however, there is no complex study detecting both viral genetic material and viral proteins in separated cell subpopulations, as well as determining the frequency of cell infection with HCV. While presence of negative strand HCV RNA indicates virus RNA replication, NS3 HCV production indicates the presence of viral mRNA translation and polyprotein processing [23]. Non structural protein (NS3) as an essential component of the HCV replication life cycle, and plays an important role in the pathogenesis of HCV.

The aim of the present study was to determine the prevalence of HCV infection in separated PBMC fractions: T cells (CD3^+^), B cells (CD19^+^) and monocytes (CD14^+^). The presence of HCV intermediate replication product (negative strand RNA) as well as the presence of viral NS3 protein were analyzed in different cell subpopulations.

## Results and discussion

The mechanisms of HCV replication in cells of the immune system are far from clear. Invasion of cells by this virus may be consequential as previous *in vivo* and *in vitro* studies showed modified multiple gene expression and apoptotic pathways in HCV infected cells [14,25,26]. Thus, it is important to recognize the principal target population for HCV within cells of the immune system and further determine the role of the virus in cell dysfunction, particularly in the light of the were described detrimental effect of HIV/HCV coinfection [14].

Previous studies have reported evidence for extrahepatic replication of HCV in peripheral blood mononuclear cells (PBMCs) [2-7]. However, discrepancies exist regarding the main cell populations infected [8,13,21,23]. Other studies have reported evidence for HCV replication in granulocytes and dendritic cells as well as in extrahepatic tissues [23]. While extrahepatic replication is gaining wide acceptance some authors consider it to be controversial [27,28].

Replicative forms of HCV were detected in all studied subpopulations: CD3^+^, CD14^+^, CD19^+^.

The negative strand HCV RNA was detected in 7/26 (27%) PBMC samples with similar frequency in all separated CD3^+^, CD14^+^ and CD19^+^ cells, 3/26 (11.5%), 2/26 (7.6%) and 4/26 (15.3%) respectively (Table 1). The presence of genomic (positive) HCV RNA strand was detectable in all cell specimens studied (data not shown). The measurement of positive to negative strand ratio was not performed due to the limited amount of material and low viral loads. However, our previous studies indicated that the genomic RNA strand is typically detectable at a level 1–2 logs higher than the negative strand [29].

**Table 1:** Demographic, clinical and laboratory data of the patients

**No.**	**Sex**	**Age (years)**	**ALAT (IU/l)**	**GGTP (IU/l)**	**Hist-pat**	**Cryoglob.**	**Genotype**	**Negative strand HCV RNA**			**NS3 positive cells (%)**		
								**CD3**^**+**^	**CD14**^**+**^	**CD19**^**+**^	**CD3**^**+**^	**CD14**^**+**^	**CD19**^**+**^
1	F	61	31	53	G9, S2	**+**	1b	**-**	**+**	**-**	17	**-**	-
2	M	52	35	nd	nd	-	1b	**-**	**-**	**-**	**-**	-	**-**
3	M	49	55	28	Cirrhosis	-	1b	**-**	**-**	**-**	8	**-**	-
4	M	59	81	58	G4, S3	-	1b	**-**	**-**	**-**	**-**	-	**-**
5	F	66	36	54	Cirrhosis	**+**	1b	**-**	**-**	**-**	7	1	1
6	F	63	55	29	G4, S2	**+**	1b	**-**	**-**	**+**	**-**	-	**-**
7	F	69	63	157	nd	**+**	1b	**-**	**-**	**-**	4	-	-
8	F	59	77	121	nd	-	1b	**-**	**-**	**-**	**-**	-	**-**
9	F	54	211	39	nd	-	1b	**+**	**-**	**-**	1	-	2
10	F	51	64	81	G9, S2	**+**	1b	**+**	**-**	**-**	**-**	-	**-**
11	M	50	50	85	Cirrhosis	**+**	3a	**-**	**-**	**-**	1	-	-
12	F	59	88	37	G5, S2	**+**	1b	**-**	**-**	**-**	**-**	2	4
13	F	54	71	91	Cirrhosis	-	1b	**-**	**-**	**-**	**-**	-	**-**
14	F	62	116	64	G5, S2	**+**	1b	**-**	**+**	**-**	2	2	1
15	F	58	158	58	G8, S2	-	1b	**-**	**-**	**-**	**-**	-	**-**
16	M	58	35	133	G7, S2	-	1b	**-**	**-**	**-**	**-**	2	**-**
17	F	62	26	97	Cirrhosis	-	1b	**-**	**-**	**-**	9	**-**	23
18	M	52	2165	401	acute h.	-	1b	**-**	**-**	**-**	5	1	-
19	M	30	147	142	G8, S2	-	1b	**-**	**-**	**+**	**-**	-	**-**
20	F	64	307	140	Cirrhosis	**+**	1b	**-**	**-**	**-**	**-**	-	**-**
21	F	58	63	109	Cirrhosis	-	1b	**-**	**-**	**-**	8	-	-
22	F	65	79	24	G4, S4	**+**	1b	**-**	**-**	**-**	**-**	-	1
23	M	34	90	54	G5, S1	-	1b	**-**	**-**	**-**	1	1	-
24	M	28	105	28	G5, S1	-	3a	**-**	**-**	**-**	**-**	**-**	**-**
25	F	42	44	80	Cirrhosis	-	1b	**+**	**-**	**-**	**-**	**-**	**-**
26	F	63	26	nd	nd	**+**	1b	**-**	**+**	**-**	**-**	**-**	**-**

Note: F, female; M, male; positive result (+); negative result (−); nd - not done; cryoglobulinemia (cryoglob.), histopatological finding (hist-pat.); G, grading (0–18); S, staging (0–4); (according to modified Knodell Score); [34]. Normal values of enzymes: ALAT (alanine aminotransferase): 10–52 F and <70 M; GGTP (gamma-glutamyltransferase): <43 F and < 73 M.

The presence of HCV NS3 protein was observed in more than half 15/26 (57%) of the examined PBMC samples, most frequently in CD3^+^ cells: 11/26 (42.3%), followed by CD14^+^ and CD19^+^ cells: 6/26 (23%). The observed prevalence of HCV infection was higher than reported in other studies performed in immunocompetent patients, probably due to the application in the current analysis of two different viral replication markers [3,7,13].

Previous studies showed that CD3^+^ cells harboured the virus more often than the remaining PBMC subpopulations [3,14,15,19,30].

**Figure 1:**
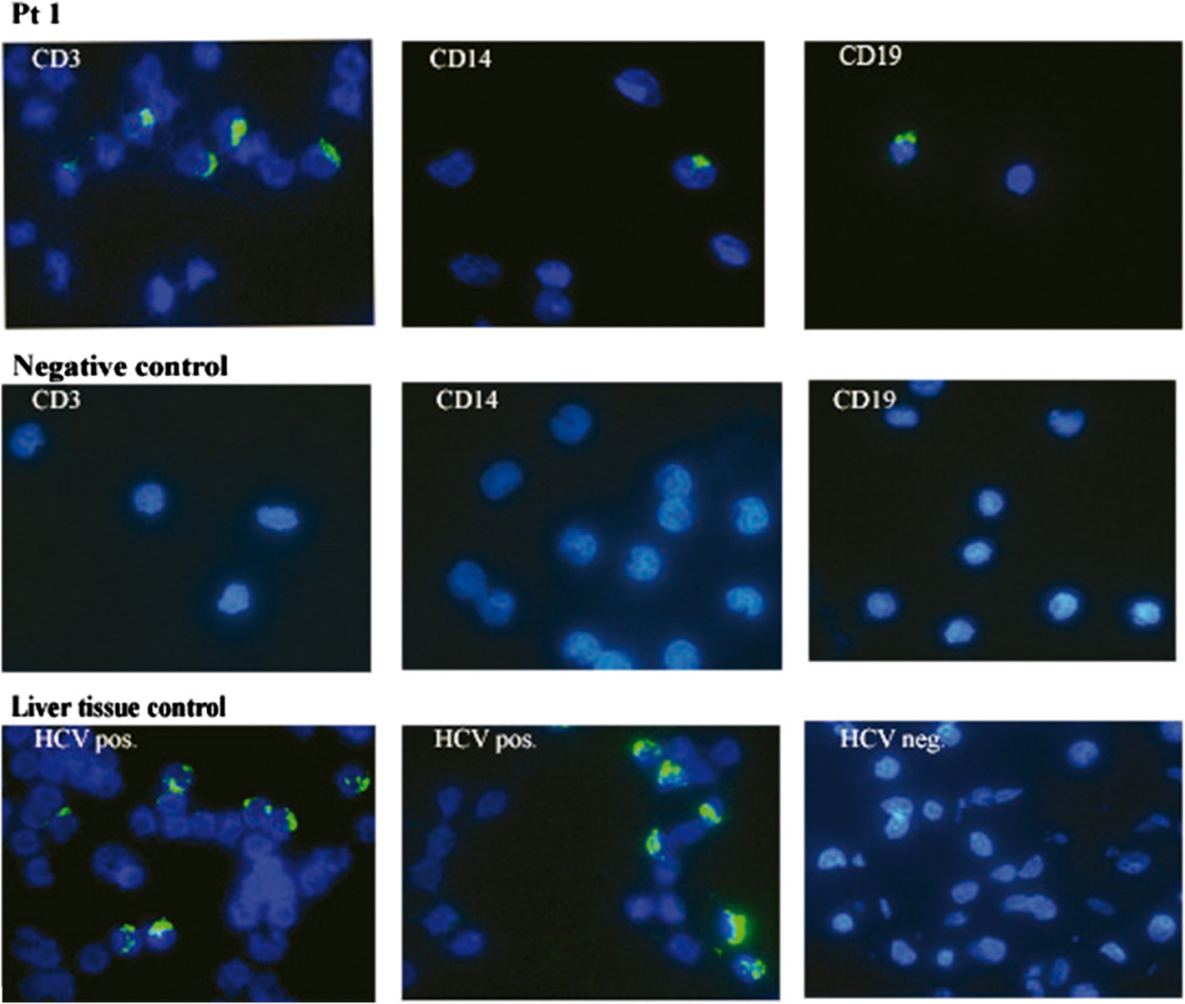
**Detection of NS3 HCV in PBMC subpopulations: CD3^+^, CD14^+^ and CD19^+^ (Pt 1).** Negative control CD3^+^, CD14^+^ and CD19^+^ subpopulations were collected from HCV - negative patient. Liver tissue control: liver biopsy samples from HCV positive patient (HCV pos.) and HCV negative patient (HCV neg.).

In our study we found that half of CD3^+^ samples (13/26) were NS3 HCV positive, whereas in CD14^+^ and CD19^+^ the prevalence reached 30.8%. In two patients, NS3 HCV was observed in all three examined cell subpopulations (Table 1; Figure 1).

Proportion of NS3 positive cells within cell subsets was highest for CD3^+^ cells (range: 1–17; mean: 2.4 ± 4.2), whereas for CD14^+^ (range: 1–20; mean: 4 ± 0.7), and CD19^+^ (range: 1–23; mean: 1.2 ± 4.5); (Table 1).

No correlation between NS3 HCV and HCV RNA negative strand was found, which could be due to low level of the virus replication [5,24]. While in some other studies the presence of HCV proteins was correlated with HCV RNA, however, this was based on the analysis of the genomic, not negative strand RNA [1,8,31,32].

The prevalence of NS3 - positive cells is lower than observed in the liver were proportion infected cells varies from 0.04% to more than 80% [31,33]. Results of the current study are similar to those published by Rodriguez-Inigo et al. who used *in situ* hybridization in unselected PBMC. In that study infected cells constituted 0.08% to 4% of all PBMC [8].

## Conclusions

Our results indicate that CD3^+^ cells are a dominant site for extrahepatic HCV replication, although other PBMC subpopulations may also support virus replication.

## Methods

Blood samples were collected on EDTA, from 26 consecutive chronically HCV infected patients (male/female 17/9; mean age 54.69 ± 8.12) treated at Clinic of Hepatology and Acquired Immune Deficiencies, Medical University of Warsaw.

Twenty five patients were infected with genotype 1b, one with genotype 3a.

Eleven patients (42%) were diagnosed with crioglobulinemia. Demographic and clinical characteristics of the studied patients are shown in Table 1[34].

The study was approved by the Internal Review Board at the Warsaw Medical University (ref. no. KB/17/2013) and each patient provided a written consent.

### Magnetic cells sorting

PBMC were isolated by density gradient centrifugation on Histopaq (Sigma, Missouri, USA) from 10 ml of blood collected into a tube containing EDTA. The PBMC subpopulations: CD3^+^, CD14^+^, CD19^+^ were isolated by positive selection by IMag (magnetic cell separation system, BD IMagnet™; San Jose, USA). For separation of particular cell subpopulations, the following antibodies were used: anti-CD3 (anti-human CD3 magnetic Particles-DM, BD IMag™, San Jose, USA), anti-CD19 (anti-human CD19 magnetic Particles-DM, BD IMag™; San Jose, USA), anti-CD14 (anti-human CD14 magnetic Particles-DM, BD IMag™; San Jose, USA).

Purity of each population was verified by flow cytometry (Canto II, BD) using the antibodies: PE mouse anti-human CD3 (BD Pharmingen™, San Jose, USA), PE mouse anti-human CD14 (BD Pharmingen™; San Jose, USA), APC mouse anti-human CD19 (BD Pharmingen™; San Jose, USA), and isotype control: PE and APC mouse IgG1ĸ (BD Pharmingen™; San Jose, USA).

**Figure 2:**
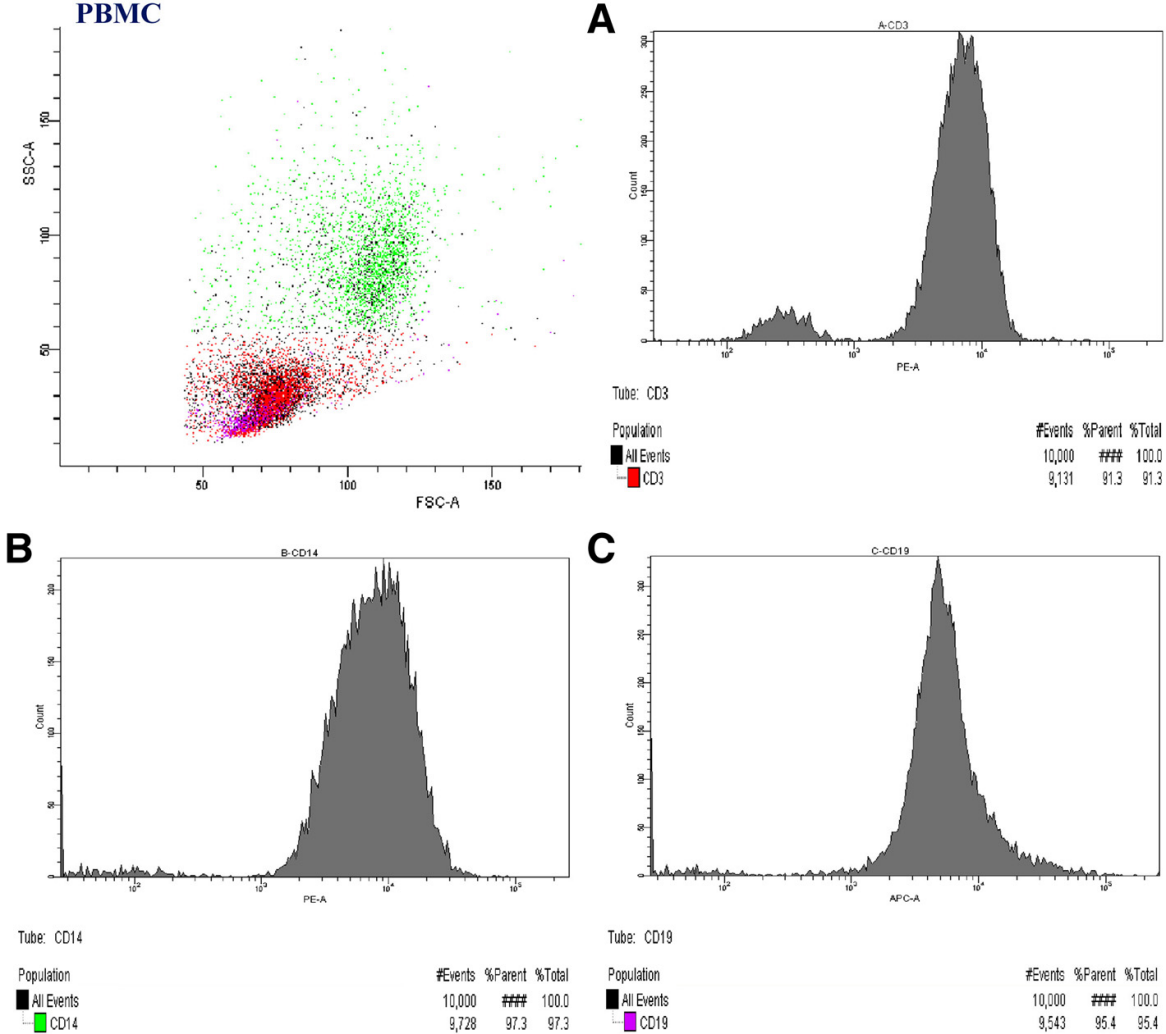
The purity of CD3^+^(A), CD14^+^(B) and CD19^+^(C) cell subpopulations separated from PBMC collected from HCV positive patient.

The purity of the separated CD3+, CD14+ and CD19+ cells was no lower than 92.4%, 97.5% and 96.7%, respectively (Figure 2).

### Detection of negative strand HCV RNA

HCV RNA was extracted from 1×10^6^ PBMC and 2-3×10^4^ of separated CD3^+^, CD14^+^and CD19^+^ subpopulations, as described elsewhere [1]. Qualitative detection of the negative-strand HCV RNA, spanning 5’UTR viral region was accomplished by specific synthesis of cDNA in 70°C using the thermostable enzyme *Tth* (Applied Biosystem, Life Technologies, Carlsbad, USA).

For Tth-based RT-PCR detection of the negative strand, cDNA was synthesized in 20 μl of reaction mixture containing 50 pM sense primer, 1x RT buffer (Applied Biosystem, Life Technologies, Carlsbad, USA), 1 mM MnCl2, 200 mM (each) dNTP, and 5 U of Tth (Applied Biosystem, Life Technologies, Carlsbad, USA). After 30 min at 72°C, Mn^2+^ was chelated with 8 μl of 10x EGTA chelating buffer (Applied Biosystem, Life Technologies, Carlsbad, USA), 50 pM antisense primer was added, the volume was adjusted to 100 μl, and the MgCl concentration was adjusted to 2.2 mM. To increase the specificity and sensitivity of assay, wax beads (Ampliwax; Applied Biosystem) were routinely employed for “hot start”. The amplification was performed in Perkin Elmer GenAmp PCR System 9600 thermocycler (Perkin Elmer, Waltham, Massachusetts, USA). The sequence of primers and reaction conditions are shown in Table 2. The PCR products were analyzed on 2% agarose gel.

**Table 2:** Sequences of primers and PCR conditions

	**Sequences of primers**	**PCR conditions**
**Reverse transcription**	5′-TGR TGC ACG GTC TAC GAG ACC TC-3′	Temp. 72°C/30 min
**I PCR**	5′-RAYCACTCCCCTGTGAGGAAC-3′	1 min at 94°C;
		50 cycles of 94°C for 15 s, 58°C for 30 s, and 72°C for 30 s; 72°C for 7 min
**II PCR**	5′-ACTGTCTTCACGCAGAAAGCGTC-3′	1 min at 94°C;
	5′-CAAGCACCCTATCAGGCAGTACC-3′	30 cycles of 94°C for 15 s, 58°C for 1 min, and 72°C for 1 min; at 72°C for 7 min

All PCR runs included positive controls, consisting of end point dilutions of the respective RNA strands. The titers were determined by analyzing 10-fold serial dilutions of the template. To prevent contamination, pre-PCR steps were carried out in separate room. Negative controls consisting of PBMC from HCV negative patients were included in all reaction series: one negative sample was processed for every 3 to 4 tested specimens. Under these conditions, none of the negative controls was positive [35].

The strand specific assay used in the study was capable of detect 100 viral genomic eq/ml of the correct negative strand or HCV genomic strand, while ≥ 10^8^ genomic equal of the incorrect strand. The amount of used RNA was 1–4 μg RNA per reaction [5].

The results were reported as positive or negative.

### Detection of HCV NS3 in PBMC and separated cell subpopulations

PBMC smears preserved in acetone/chloroform were incubated with primary monoclonal anti-NS3 antibody (Novocastra™, Leica, Newcastle, UK), following incubation with Alexa 488 goat anti-mouse secondary antibody (Life Technologies, Carlsbad, USA). To stain the cell nuclei, preparations were incubated with Hoechst reagent (Molecular Probes, Carlsbad, USA) and analyzed by fluorescent microscope Eclipse 80i (Nikon, Tokio, Japan) using 330–380 nm (Hoechst) and 450 – 490 nm (Alexa 488) UV-2A filter.

The control material included PBMC samples collected from uninfected patients and liver biopsy samples from HCV infected patients (positive samples) and uninfected patients (negative control). The specificity of the method was verified by an “internal” reaction control without a primary antibody (Figure 1).

### Statistical analysis

Statistical analysis was performed using one-way analysis of variance (ANOVA) and chi square test (Install 07). Spearman’s correlation test was used to investigates the linear relationships between presence of NS3 HCV in individual cell subpopulations: CD3^+^, CD14^+^, CD19^+^, and negative strand level HCV RNA. *P* values > 0.05 was considered to be statistically significant. The analysis was performed by use of Statistica software, version 10.

## Competing interests

The authors declare that they have no competing interests.

## Authors’ contributions

AP for study design, laboratory work (immunohistochemistry, magnetic cell sorting flow cytometry), statistical analysis, interpretations of results and preparation of manuscript, NK for laboratory work (immunohistochemistry, magnetic cell sorting, flow cytometry), JJ for sample collection, analysis and interpretations of results analysis and interpretations of results, IBO for analysis and interpretations of results, KC for analysis and interpretations of results, MF for cell preparation, detection of negative strand HCV RNA. TL for interpretations of results preparation of manuscript, MR for study design, analysis and interpretations of results and preparation of manuscript. All authors read and approved the final manuscript.
